# Current understanding of the impact of climate change on mental health within UK parliament

**DOI:** 10.3389/fpubh.2022.913857

**Published:** 2022-09-16

**Authors:** Lucy T. Pirkle, Neil Jennings, Ans Vercammen, Emma L. Lawrance

**Affiliations:** ^1^Department of Brain Sciences, Imperial College London, London, United Kingdom; ^2^Grantham Institute – Climate Change and the Environment, Imperial College London, London, United Kingdom; ^3^Centre for Environmental Policy, Imperial College London, London, United Kingdom; ^4^The School of Communication and Arts, The University of Queensland, St Lucia, QLD, Australia; ^5^Institute of Global Health Innovation, Imperial College London, London, United Kingdom; ^6^Mental Health Innovations, London, United Kingdom

**Keywords:** mental health, climate change, UK parliament, anxiety, flooding, eco-anxiety, climate policy

## Abstract

There is growing evidence that climate change is linked to adverse mental health outcomes, with both direct and indirect impacts already being felt globally, including within the United Kingdom (UK). With the UK parliament tasked with passing legislation to mitigate against and adapt to climate change, it is well placed to take a lead in implementing policies that reduce the impact of climate change on mental health and even provide mental health benefits (e.g., by increasing access to green space). The extent to which the UK parliament considers the relationship between climate change and mental health in its decision-making was previously unknown. We report, through quantitative thematic analysis of the UK Hansard database, that the UK parliament has only infrequently made links between climate change and mental health. Where links have been made, the primary focus of the speeches were around flooding and anxiety. Key mental health impacts of climate change reported in the academic literature, such as high temperature and suicides, or experiences of eco-anxiety, were found to be missing entirely. Further, policies suggested in UK parliament to minimise the impact of climate change on mental health were focused on pushing adaptation measures such as flood defences rather than climate mitigation, indicating potential missed opportunities for effective policies with co-benefits for tackling climate change and mental health simultaneously. Therefore, this research suggests a need to raise awareness for UK policymakers of the costs of climate inaction on mental health, and potential co-benefits of climate action on mental health. Our results provide insight into where links have and have not been made to date, to inform targeted awareness raising and ultimately equip policymakers to protect the UK from the increasingly large impacts of climate change on mental health.

## Introduction

Climate change has been posed as the “biggest global health threat of the 21st century” (1). Research and action on the intersection between climate change and human health has so far focused predominantly on physical health, mirroring the historic trends where mental health is “the most neglected of all human conditions” (2). However, there is increasing evidence for a range of significant links between mental health and a changing climate, resulting both directly and indirectly. With the impacts of climate change increasingly evident in the UK and globally, it is essential that the significant but often hidden and unaccounted for impacts on mental health and emotional wellbeing are considered by those who have the power and influence to mitigate against and respond to the threats.

Climate change is believed to have widespread effects, including shifts in global temperature and weather patterns. Increases in average global temperatures and extreme weather events have both been shown to directly impact mental health. Rising global temperatures, for example, have been linked to higher suicide rates (3), greater hospitalisations for mental disorders (4, 5) and worse population mental health (6). Suicide rates, for example, have been shown to increase with ambient temperatures, with some evidence also for an effect of heatwaves. Page et al. (3) report a 49.6% increase in suicides during the 1995 heatwaves across England and Wales, with relative risk increasing with every degree increase in ambient temperature (3). Given the UK's ten warmest years on record have all occurred since 2002 and heatwaves are now 30 times more likely to happen due to climate change (Met Office) (7), understanding and managing the impacts on mental health will be crucial.

Similarly, extreme weather events including wildfires (8), floods (9, 10), droughts (11) and severe storms (12) have all been linked to adverse mental health outcomes globally. The UK has been particularly vulnerable to flooding (13), with large flooding episodes becoming more frequent in the UK under a changing climate. Flooding increases the risk of mental health disorders in exposed individuals (13). For example, the Public Health England commissioned English National Study of Flooding and Health report stated that the winter 2013–14 UK floods significantly increased probable depression, anxiety and post-traumatic stress disorder (PTSD) in affected individuals, even 2 years after the floods (9). With increased risk of multiple exposures to extreme weather events, appropriate UK government policy and planning for mitigating the risk of flooding on mental health and responding to increased mental health needs before, during and after flood events is essential to minimise the impact on individuals and communities.

Climate change has also been shown to indirectly impact mental health and emotional wellbeing (14). A range of affective responses, collectively termed “eco-emotions”, are linked to increased awareness of the growing crisis and the effect it will have on society and the environment (15). Negative eco-emotions are becoming increasingly prevalent in the UK and globally, with negative climate-related emotions found to be present in all 25 countries explored by Ogunbode et al. (16), including the UK. This study also highlighted that negative eco-emotions was related to both insomnia and lower self-rated mental health, highlighting the potential implications of climate-related distress for mental health and indicating this could be a considerable burden as climate change worsens in the UK and globally. Certain groups, such as children and young people, are thought to be at a heightened risk of negative eco-emotions (17, 18). Although these “eco-emotions” are understandable and can be even healthy responses to the experienced threats and losses, and are not in themselves clinical conditions, severe cases can require medical intervention (19), especially if they exacerbate other mental health disorders such as anxiety, depression or insomnia (16, 20), and there are reports of distress being so severe that the young people affected become suicidal (21).

### The importance of UK parliament in this issue

Given that there is growing risk to mental health both directly and indirectly from climate change, it is important that people with power and influence are working to mitigate against and respond to those threats. There has been significant legislation passed by the houses of UK parliament on both climate change and mental health as separate critical challenges. The Mental Health Act was passed in 2007 and the Climate Change Act was passed in 2008, with amendments such as Net Zero 2050 made more recently (22–24). However, it is unclear to what extent UK decision-makers are considering the intersections between these critical issues and whether discussions are framed with reference to synergistic policy solutions.

One key group who have the potential to protect population mental health from the impacts of climate change, is UK parliamentarians. This group have the ability to pass legislation, which protects people from the negative impacts of climate change on mental health. For legislation to be passed, it must be debated and scrutinised within parliament first. Therefore, parliamentary debates provide insight into the opinions and policy positions of parliamentarians and therefore can set the scene for future legislation. Once legislation is drafted on the back of these debates, approval decisions usually require support from both houses of parliament, i.e., the House of Commons and the House of Lords.

The roles of both Houses in drafting and approving legislation, holding the government accountable, and in policy debate are largely similar. However, a significant difference between the Houses is the way in which members are chosen. The House of Commons holds 650 seats democratically elected by the public, with members known as members of parliament (MPs). The political party with the most elected seats forms the government. The two largest parties in the Houses of Commons are the Conservatives and Labour who, as of this study in 2021, had 56 and 31% of the seats, respectively, following the 2019 general election. The ~800 members of the House of Lords can be appointed for a number of reasons and are known as “life peers”. Appointees include: MPs who are appointed life peers at the end of their tenure in the House of Commons, former speakers of the House of Commons, one-off appointees for government ministers who are require a seat in one of the Houses of Parliament but are not an elected MP, or individuals who are appointed in “resignation honours” by a Prime Minister upon their resignation. Life peers also include 26 Church of England Archbishops and bishops. 33% of life peers are part of the Conservative party and 22% are Labour. Non-affiliates and bishops do not belong to any parliamentary group.

Once legislation has support across both the House of Commons and House of Lords, it can be passed and associated policies can be enacted to deliver the proposed benefits to UK citizens. Obtaining such support is important for a number of reasons. Firstly, parliament has the power to ensure appropriate mental health support is available to individuals and communities experiencing climate distress and therefore averting escalating mental health needs. This could include investing in actions to increase resilience, or one's ability to adapt to the stresses of climate change (25). Research has suggested that through increasing community cohesion and encouraging community action against climate change, individuals are less likely to develop climate-related mental health disorders (26). By beginning to build emotional resilience within communities early, future demand for mental health services as climate change progresses may be decreased. Additionally, policies could be designed to deliver benefits to population mental health which extend further than just reducing the negative impacts of, for example temperature rises (27). Policies such as the development of green and blue spaces in urban areas to provide equitable access to nature, active transport such as more opportunity to walk or cycle over driving, improving home energy efficiency, and cutting air pollution can all reduce emissions of greenhouse gases whilst synergistically having a positive effect on mental health (25). For example, increased access to green spaces in otherwise urbanised communities, allows greater opportunity to connect with and benefit from time in more natural settings.

Given the importance of parliament debating this topic for effective legislation to be passed, this study aims to understand the attention given to the relationships between climate change and mental health in UK parliamentary discussions. This study represents the first known analysis of relevant parliamentary records, to understand when, by whom, in what contexts, and to what end, mental health and climate change were being considered together. By analysing UK parliamentary discussions from the Hansard database, the objectives of this research are to identify:

a) The extent to which the interconnections between climate change and mental health have been considered, and how this has changed over time;
b) The contexts in which these issues were discussed, including by whom the specific instance was raised (politicians and associated political parties), and which particular policy direction (if any) the instance supported;
c) The specific impacts of climate change on mental health discussed by parliamentarians to ascertain whether the full spectrum of impacts have been considered;
d) Which groups of society have been recognised as affected by the mental health impacts of climate change.

## Methods

### Search strategy

To analyse discussions on climate change and mental health in UK Parliament, we conducted a targeted search of the Hansard database (https://hansard.parliament.uk/search/)—a government repository of transcripts from all parliamentary debates across both the House of Lords and House of Commons.

To gauge the general trend in discussions within the UK parliament on climate change and mental health as separate issues over the past 25 years, we searched the Hansard database for the terms “climate change” and “mental health”. The search covered the period 1st January 1995 to 31st December 2020, and allowed for a yearly breakdown of the number of speeches referencing each issue, as well as the total number recorded across the 25-year period. The total annual number of parliamentary speeches was also obtained from Hansard.

Next, we searched Hansard across the years from 01/01/2001 to 31/12/2020. To identify speeches where relevant links between climate change and mental health were discussed, multiple search terms were chosen to cover significant key words for each of the two topics (Table 1). The climate change-related terms were chosen based on all climate change-related weather events listed on the UK Meteorological Office website. A core list of key terms has not yet been published for mental health risks of climate change. Therefore, the mental health terms were chosen based on a review of relevant literature to identify all mental health and emotional wellbeing terms commonly related to climate change. These included standard mental health terms and those developed or emerging to capture more climate and environment-specific mental health and emotional wellbeing terms, sometimes referred to as “planetary mental health terms”. Minor variations in the terms were also included. Each mental health term was searched in combination with each climate change term generating 286 unique searches. For the planetary mental health specific terms (eco-anxiety, ecological grief, and solastalgia), the terms were searched alone without a corresponding climate change term as these terms already link the two topics.

**Table 1:** Search terms used for querying the Hansard database, based on Met Office climate change-related events and literature on the links between climate and mental health.

**Mental health terms**	**Climate change terms**
Mental health	Climate change
Mental illness	Climate crisis
Depression	Global warming
Anxiety	Sea level rise
Grief	Sea level rise/rising sea levels
Post-traumatic stress disorder/PTSD	Drought/droughts
Suicide/suicides/suicidal	Flood/floods/flooding
Guilt	Storm/storms
Eco-anxiety	High temperature/high temperatures
Ecological grief	Heatwave/heatwaves
Solastalgia	Bushfire/bushfires
	Wildfire/wildfires
	Extreme weather
	Cyclone/cyclones
	Hurricane/hurricanes

Each mental health term was combined with each climate change term (including variations). Eco-anxiety, ecological grief and solastalgia were searched in isolation as by definition these already link the two themes.

### Review process

**Figure 1:**
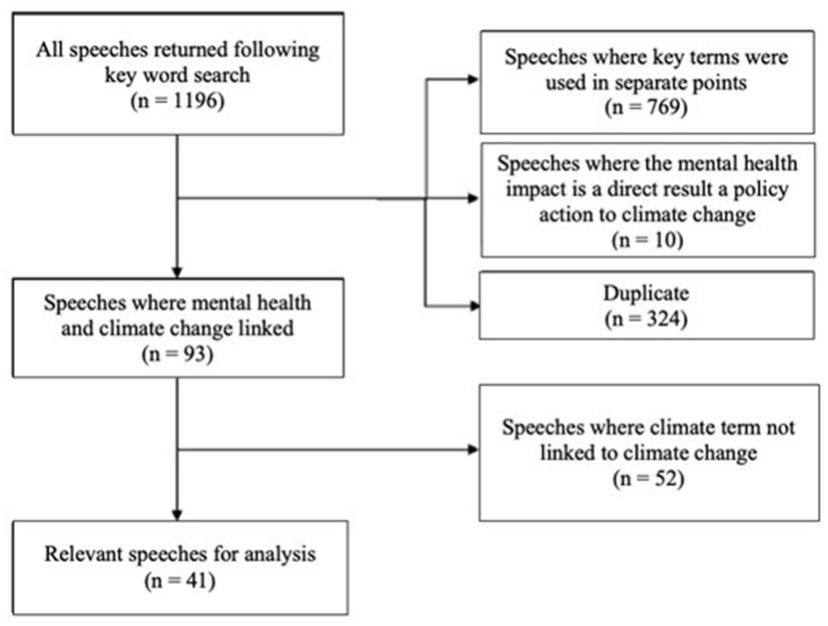
Flow diagram outlining the identification of relevant speeches where climate change was linked to mental health. Duplicate speeches returned in multiple different searches as they contained multiple keywords were removed.

All speeches returned for each search were screened for relevance and duplicates were removed (Figure 1). The speeches returned were read in isolation from the full debate, with a single speech defined by one parliamentarian speaking without interruption. Speeches were included if the speaker made reference to the intersections between climate change and mental health (e.g., a flood explicitly linked to climate change has caused anxiety). Speeches were excluded if the key words were used in separate contexts within the speech and no direct link was made between the two themes. This includes, for instance, a particular climate change policy causing anxiety (e.g., due to the development of large wind farms near homes) rather than climate change *impacts* causing such anxiety. Speeches were also excluded if an extreme weather event was linked to mental health without directly mentioning how the extreme weather event relates to a changing climate.

### Analysis

For each relevant speech the following information was recorded: speech date, House where the speech took place (Commons or Lords), political party of the speaker and any parliamentary position held by the speaker.

The content of the speech (speech text) was then analysed using a quantitative thematic approach, following a set of questions corresponding to the predetermined research objectives, and codes were assigned as outlined in Table 2 (28). The first coder (LP) coded all relevant speeches, and 25% were independently coded by a second coder (NJ) to ensure consistency. Cohen's Kappa interrater reliability was calculated on STATA/BE v17.0 and indicated substantial agreement between raters (κ = 84.21 across codes). Once codes were assigned, the numerical codes were analysed quantitatively.

**Table 2:** Codes used for thematic analysis of relevant speeches.

**Question**	**Codes**	**Description**
What aspect of climate change is being discussed?	See Table 1	A code was assigned if any climate change term was mentioned within the speech. Multiple codes could be assigned within the same speech.
What aspect of mental health is being discussed?	See Table 1	A code was assigned if any mental health term was mentioned within the speech. Multiple codes could be assigned within the same speech.
Is the speaker calling for policy action to mitigate climate change,	1) Mitigation	The speaker's proposed policy action aims to combat the causes of climate change, e.g., reduce greenhouse gas emissions
adapt to climate change or both?	2) Adaptation	The speaker's proposed policy action is to respond to the effects of climate change to reduce additional suffering, e.g., home flood defences or increased funding for mental health
	3) Both	Reference to both proposed adaptation and mitigation policy actions in the same speech
	4) Neither	No reference to either proposed adaptation and mitigation policy actions
Do the described impacts result from the direct or indirect impacts	1) Direct	Reference is made to the direct effect of climate change or related events on mental health
of climate change?	2) Indirect	Reference is made to the indirect effects of climate change on mental health
	3) Both	Some reference to both indirect and direct effects are made
Who is identified as being affected?	1) Not specific	No specific group is identified and the effects suggested as widespread
	2) Young people	Young people are explicitly mentioned
	3) Those exposed to extreme weather events	The residents of a particular area are identified as being most affected, e.g., residents of Cumbria following a flood
	4) Other	Other is when a different group is mentioned, e.g., farmers or environmentalists
Is it discussed in the context of	1) UK	Reference to the impact being felt by UK residents
climate change impacts in the UK	2) Global	Reference to an impact outside of the UK
or abroad?	3) Not specific	No clarity on where the impact is being felt.

This analysis allowed us to further categories and understand links made between climate change and mental health by the UK Parliament.

## Results

### Frequency of parliamentary speeches on climate change and mental health as separate topics

**Figure 2:**
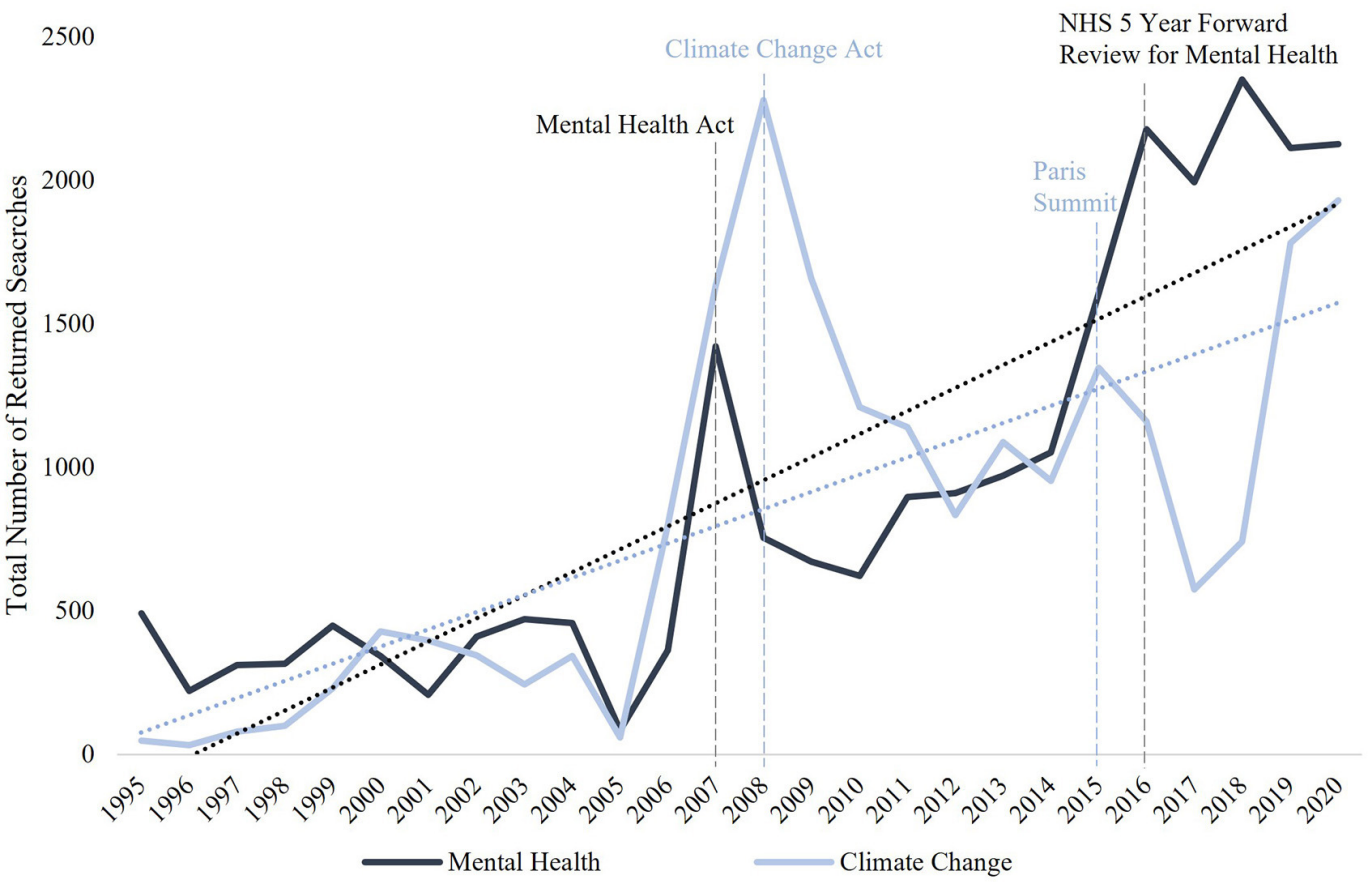
Increase in frequency of speeches around mental health and climate change within UK parliament over the past 25 years. Total number of returned searches across both the House of Commons and House of Lords was found using a search for the terms ‘climate change' and ‘mental health' for each year from 1995 to 2020 (01/01–31/12) on UK Hansard database. Key reports/events included as references. Linear trend lines show the overall trend in this change.

Trends in the frequency of UK parliamentary speeches on the separate issues of climate change and mental health over the 25 years to 2020 on UK Hansard database are shown in Figure 2. The total number of speeches recorded on Hansard per year remained relatively constant throughout these 25 years. However, after 2005, considerable increases in total mentions of both climate change and mental health as separate topics were detected, and in particular appeared to precede the passing of major relevant legislation, i.e., the Mental Health Act in 2007 and the Climate Change Act in 2008 (29). It is notable that after these major pieces of legislation for each issue passed there was a sharp reduction in parliamentary attention devoted to the issues, but with a higher baseline than previously. Similar rapid increases were also observed for climate change in the year of the COP21 Paris Summit and for mental health in the year the NHS 5 Year Forward Review for Mental Health was published (2016). In 1995, climate change and mental health were mentioned in 0.04 and 0.43% of total speeches, respectively, whereas in 2020 this increased to 1.73 and 1.91% of speeches, respectively.

### Frequency of parliamentary speeches mentioning both climate change and mental health

**Figure 3:**
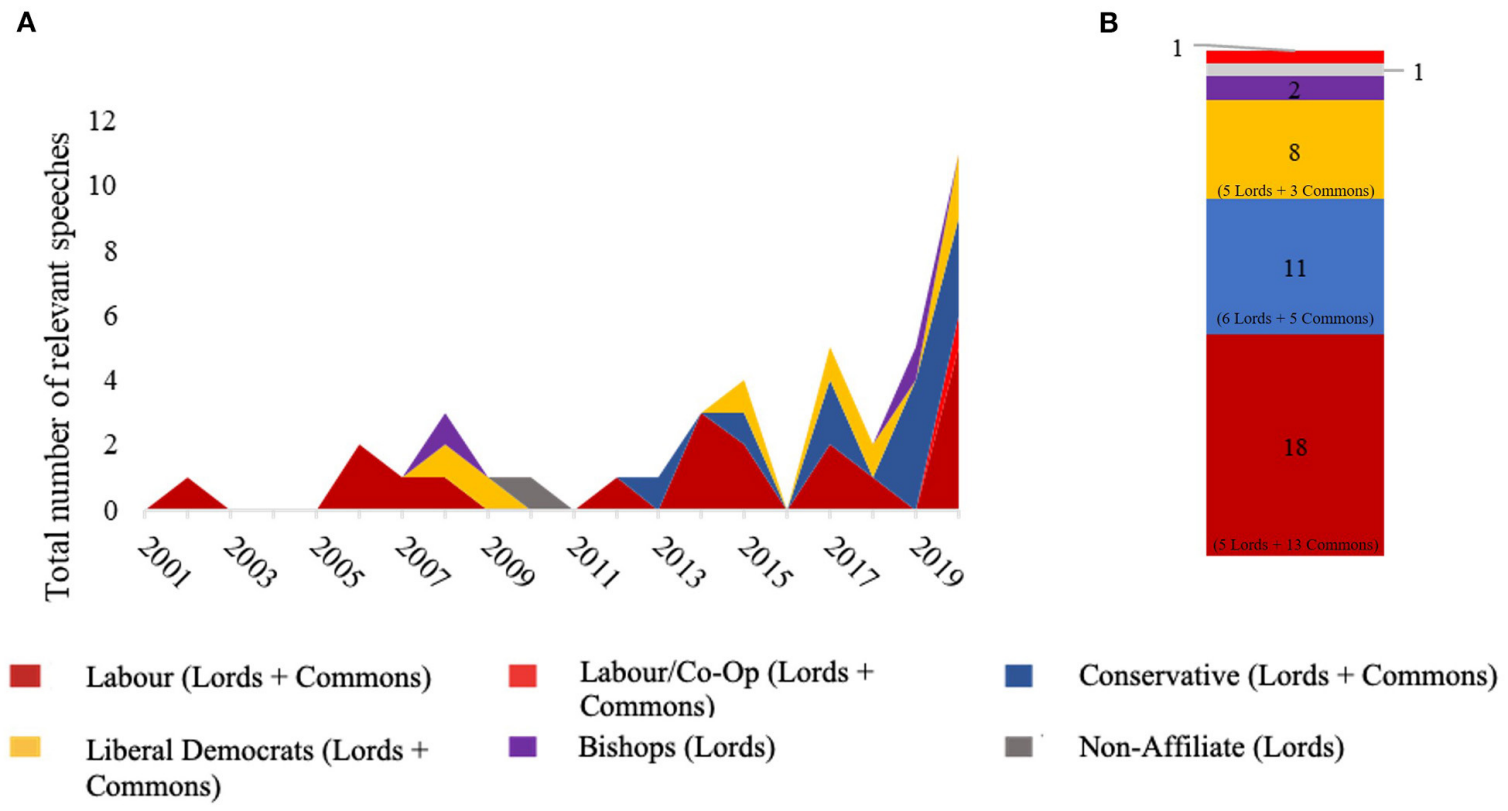
**(A)** Increase in frequency of speeches on the links between mental health and climate change with political breakdown and **(B)** total number of speeches by political party across the 20 years (01/01/2001–31/12/2020). The analysis includes only speeches where direct links between mental health and climate change were mentioned (*N* = 41).

Examining the temporal distribution of the 41 speeches where a term for mental health and for climate change were mentioned together, there has been an upward trend in frequency from 2001 to 2020, with a noticeable increase since 2018 (Figure 3A). Despite an increase in attention, in 2020 only 11 speeches referenced a link between climate change and mental health compared to 1,930 speeches that referred to climate change and 2,126 that referred to mental health separately. Of all relevant speeches linking climate change and mental health to date, most (49%) were by Labour or Labour/Co-Operative politicians who represent the political Left. However, there also is some involvement in these speeches from all major political parties, with 29% of speeches from Conservative politicians (who typically represent the political Right) and 17% from Liberal Democrats (who are a relatively centrist party) (Figure 3B) (30). Both Houses discussed this topic, with a 55–45% split between House of Commons and House of Lords, respectively.

### Contextual analysis of speeches linking climate change and mental health

#### What climate- and mental health-related terms are used in parliamentary discussions and how are they being linked?

**Figure 4:**
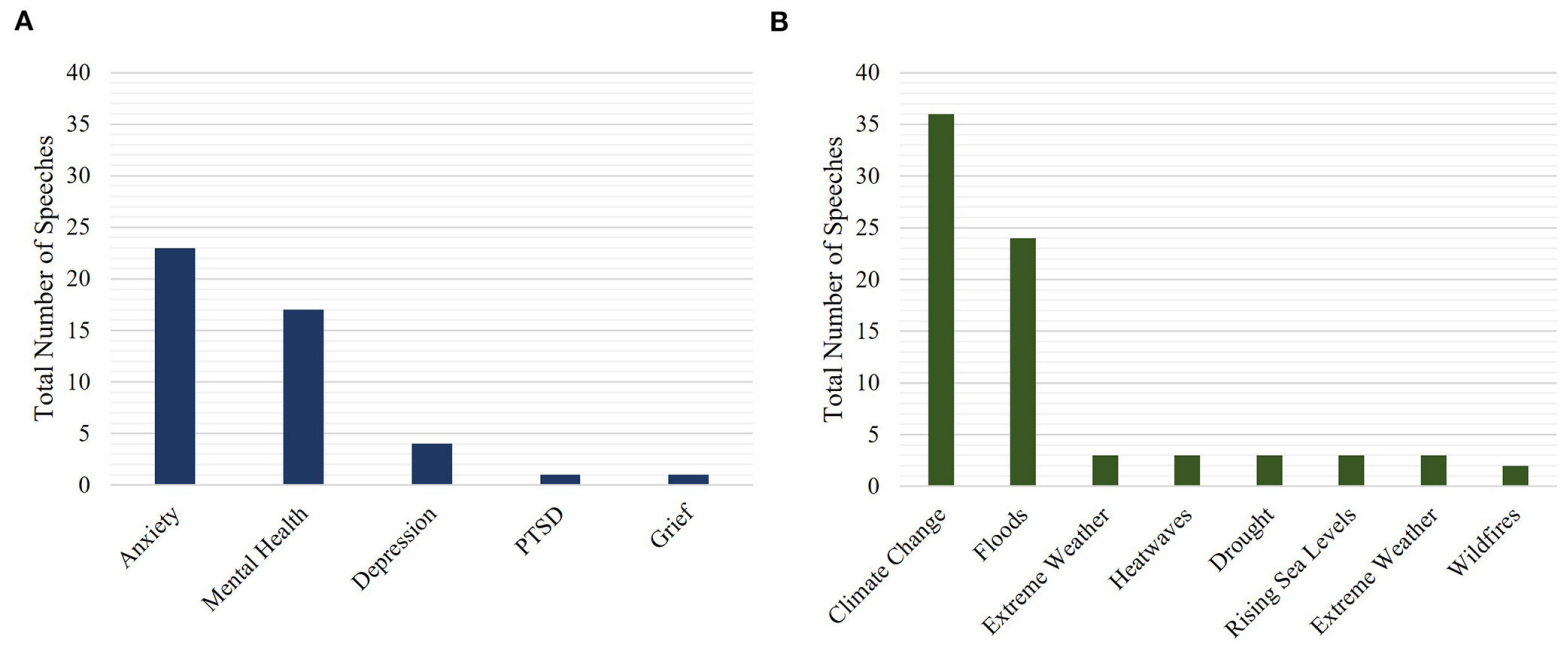
Frequency of specific terms related to **(A)** mental health and **(B)** climate change. The analysis includes only speeches where direct links between mental health and climate change were mentioned, for the time period between 01/01/2001 and 31/12/2020 (*N* = 41). Some speeches mentioned more than one key term.

Each of the identified speeches was assessed for specific references to key climate change and mental health terms to gain insight into the context in which these speeches were happening. Results show the terms “mental health” (17 mentions) and “anxiety” (23 mentions) were the most frequent mental health terms used in relevant speeches (Figure 4A). This included a speech in the House of Commons by MP Catherine West (Labour), who mentioned the following:

*The Chancellor has spoken well today of the scars that are felt by so many in society due to the triple whammy of covid, climate change and Brexit. Will he outline how he will manage to ring-fence money for mental health within the health spend? Mind, the charity, has said that phone calls have doubled, with many young people experiencing debilitating anxiety, depression and self-harm. Will he urgently look at mental health and ring-fence money for workforce changes, which are desperately needed, and for a decent revenue spend to bring mental health up into line with physical health? (Catherine West, 2020)*

The broad term “climate change” (36 mentions) and more specific mentions of the term “flood” (24 mentions) were the most common climate change terms used in these relevant speeches (Figure 4B). For example, MP Tim Farron (Liberal Democrats) made the following speech in the House of Commons response to the flooding caused by Storm Desmond which hit Cumbria in the north of England in 2015:

*I was there the morning after Storm Desmond. . . Even today, there are children who still unable to sleep any time it begins to rain. . . four years of fear whenever it pours; four years of incalculable strain on mental health for the old and young alike-how dare I claim to represent them if I do not see the flood defences delivered? (Tim Farron, 2020)*.

**Figure 5:**
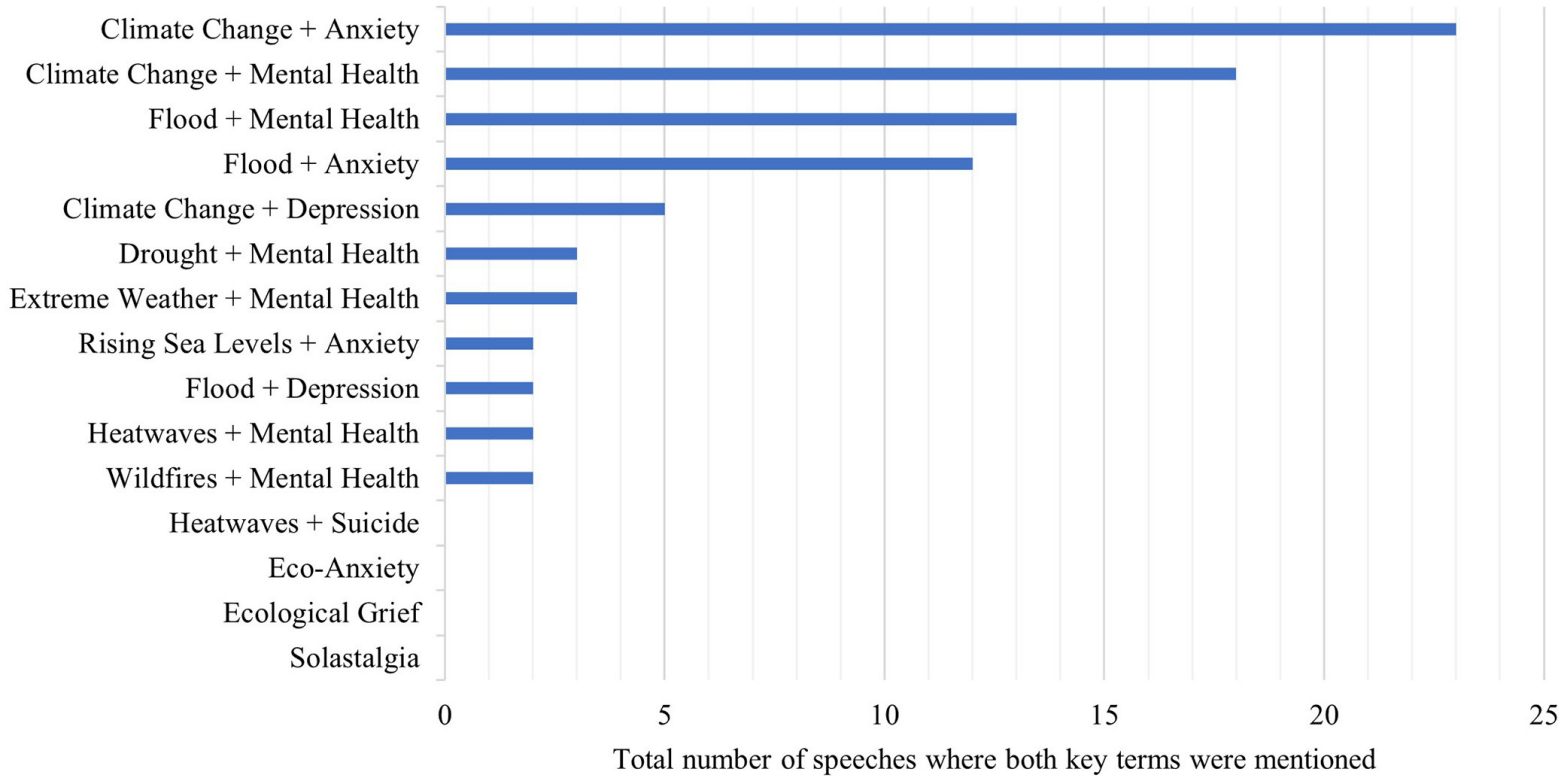
The number of speeches featuring a specific combination of mental health and climate change terms. The analysis includes only speeches where direct links between mental health and climate change were mentioned, for the period between 01/01/2001 and 31/12/2020 (*N* = 41).

We then examined which combinations of key terms were used most frequently together, within the same speech. The most common combination of terms was “climate change” and “anxiety” (23 co-mentions; Figure 5), followed by “mental health” and “climate change” (18 co-mentions), “floods” and “mental health” (13 co-mentions) and “floods” and “anxiety” (12 co-mentions). For example, Dr. Thérèse Coffey (Conservative Party) spoke of the links between flooding and mental health in the House of Commons, noting:

*I am very aware of the extreme flooding events that have been suffered and the damage that has been caused, and I recognise the impacts, including on mental health, experienced by people and communities. I pay tribute to the responders and volunteers from across the county, and indeed the country, who worked around the clock in challenging circumstances during Storm Desmond, and for their ongoing work in flood action groups (Dr. Thérèse Coffey, 2019)*.

Baroness Walmsley (Liberal Democrat) addressed the broad known links between mental health and climate change in the House of Lords:

*The natural environment contributes to our well-being and mental health, as well as our economy. For these and many other reasons, we must halt climate change and not do a Donald Trump and stubbornly deny that it has anything to do with wildfires in Florida or, of course, flooding in Yorkshire. Can we hear about the Government's progress on action against climate change? (Baroness Walmsley, 2018)*.

Anna McMorrin (Labour) also spoke of the links between anxiety and flooding in the House of Lords when she said:

*I was out in my constituency late last Friday night as the rains returned. I saw properties damaged two weeks before by the floods and people up all night, although they were not flooded again. That anxiety, worry and stress cannot be undone (Anna McMorrin, 2020)*.

The analysis revealed no mentions of specific terms used in the academic literature on planetary health, such as “eco-anxiety,” “ecological grief,” and “solastalgia.”

#### Are the mental health impacts mentioned a direct or indirect impact of climate change on different groups?

**Figure 6:**
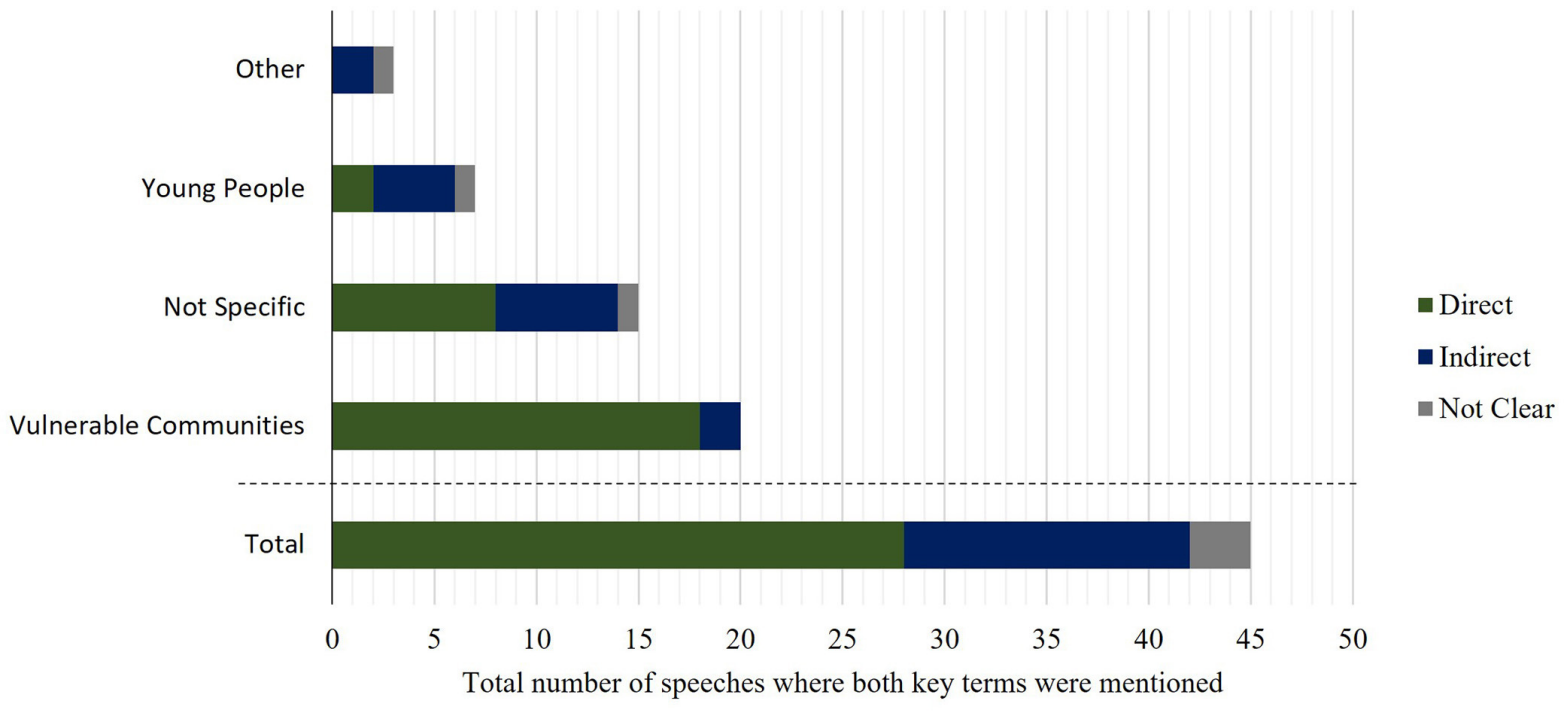
The number of speeches in which specific populations are mentioned in relation to climate change, split by whether the identified impact on the population was direct or indirect. Direct effects are defined as those where an individual experiences the effects of climate change first-hand. Indirect effects include the individual not directly experiencing the effects of climate change but being affected by knowledge of the issues. The “other” population category includes for example “those concerned by the environment”, farmers and other politicians. Some speeches mentioned more than one group. The analysis includes only speeches where direct links between mental health and climate change were mentioned, for the period between 01/01/2001 and 31/12/2020 (*N* = 41).

We then examined which populations' mental health was identified in the speeches as being affected by climate change, and whether the observed effect on mental health was direct or indirect (Figure 6). Overall, 63% (*n* = 26) of the relevant speeches represented the effects as being direct consequence of climate change. For example, Baroness Hayman of Ullock in 2017 said in the House of Commons:

*Friend the Member for Wakefield (Mary Creagh), the Chair of the Environmental Audit Committee, who talked about the huge impact that climate change is having on our communities … The flooding in Somerset was not the same as the floods on the east coast, which in turn were very different from the flooding in Cumbria… So what should we do? As has been discussed today, we need to look at the whole river catchment. We need to invest in sustainable drainage systems. And I believe that we need to stop talking about flood prevention. We cannot prevent flooding, but we can manage it and make our communities properly resilient. People are nervous and frightened, and it is time we took seriously the effect of flooding on mental health (Baroness Hayman of Ullock, 2017)*.

A further 34% (*n* = 14) referred to the indirect effects of climate change on mental health. To illustrate, George Freeman noted in a 2020 speech in the House of Commons:

*We have seen quite a lot of expertise, including from the former Chair of the Transport Committee, the hon. Member for Nottingham South (Lilian Greenwood), and I have heard an awful lot with which we agree, including on the scale of the challenge of global climate change and the imperative of gripping transport decarbonisation now. There was an important point about avoiding climate anxiety while stressing the urgency of the situation. We do not want to depress people, particularly the young, by making out that this task is impossible (George Freeman, 2020)*.

In the remaining 2% (*n* = 1) of speeches, it was unclear whether the speaker was addressing direct or indirect impacts of climate change on mental health. For example, Catherine West speech in 2019 within the House of Commons:

*In the Queen's Speech there was very little information about the climate change emergency; will he give a little more detail on what the Government will do in relation to this pressing issue that is affecting so many young people and their mental health? (Catherine West, 2019)*.

Examining the populations identified as affected by these impacts, the majority of speeches simply mentioned “vulnerable communities” (44%). Among these speeches, a higher proportion of direct effects were mentioned (90%), compared to indirect (10%). Several speeches (15%) discussed the direct impacts of flooding in Cumbria on the mental health of residents. Young people, however, were more likely to be mentioned as indirectly affected by climate change, with 57% of speeches that mentioned young people doing so in the context of the indirect effects of climate change. For example, Baroness Benjamin (Liberal Democrat) spoke in 2020 in the House of Lords:

*I have spent my entire adult life promoting the importance of the health and well-being of children and young people. As I have always said, childhood lasts a lifetime. What happens to us in childhood stays with us right through our lives and shapes us in every way, from our view of society, culture and the wider world to our wealth, health and mental well-being…Now, more than ever, the decisions we make will have colossal implications for the survival of humankind…****Every day, children and young people hear*** ***terrifying reports about climate change, global warming, war, disease and pestilence*,** ***and are having to deal with social media issues. Should we be surprised that*** ***they are feeling more and more anxious about life?*** *(Baroness Benjamin, 2020)*.

Given the global nature of climate change, speeches were assessed to see whether climate change was discussed in relation to mental health impacts occurring in the UK or internationally. The latter may be relevant to the UK's international aid work, or for preparations to support climate migrants. In 67% of the relevant speeches the focus was on UK-based impacts relevant for UK residents, while 26% of speeches considered global impacts of climate change. Global speeches included impacts on populations in other countries, for example Lord Clinton-Davis (Labour) spoke in 2007 about “*the acute anxiety that is caused to the people of Bangladesh”* by climate change.

#### Nature of proposed policy changes

The text was then analysed for suggested policy actions to minimise the impact of climate change on mental health, either by reducing climate change directly (mitigation) or providing support to prevent or respond to its impacts on mental health (adaptation). Most speeches referred to adaptive policies (49%; *n* = 20) such as increased provision of flood defences. For example, Ben Bradshaw (Labour) said in a 2012 speech in the House of Commons:

*The Environment Agency tells me that* ***upgrading*** ***Exeter's flood defences is its major priority in Devon and Cornwall—it needs to happen*,** ***and it needs to happen very quickly*. ***As we all know*, ***the trauma of flooding can be*** ***very significant indeed****. The excellent charity, the National Flood Forum, which I met yesterday to discuss the issue says: “It can take up to two years for homes to dry out and be restored and during this time, many families live in temporary accommodation. The process is stressful, time consuming and simply beyond some people, particularly those who are elderly or vulnerable*. ***The dread of flooding again*** ***can cause long term distress and mental health problems*** *(Ben Bradshaw, 2012)*.

A further 29% (*n* = 12) of the speeches suggested mitigatory policies, for example, a call to “*establish a process for managing the reduction of carbon emissions” [The Lord Bishop of Liverpool, 2008]* and “*Wiltshire declaring that it will be carbon neutral by 2030” [The Lord Bishop of Salisbury, 2019]*. Another 20% (*n* = 8) of speeches referenced a plan for both mitigating and adapting to climate change within the same speech. In the remaining 2% (*n* = 1) of speeches, it was unclear whether the speaker was advocating for adaptation to or mitigation against climate change.

## Discussion

The impact of climate change on mental health is significant and will continue to grow in coming years (8–12). If the government neglects to recognise and act on the links between climate change and mental health, society could incur significant costs. However, to our knowledge, there has been no previous examination of whether, how and to what extent these issues are being considered within parliamentary speeches.

Our results show that while mentions of the impact of climate change on mental health have increased in parliamentary speeches over the last 25 years, instances in which the links between these two pressing challenges are discussed represent a very small proportion of parliamentary debates. This suggests a concerning lack of awareness of synergies between the two issues and thus limited opportunity for adequate development of policy responses to both climate change and mental health that sufficiently consider their interactions. Specifically, there were only 41 identified speeches in UK parliament linking climate change and mental health over the last 25 years, while over 20,000 speeches have been made on each issue separately.

Our thematic analysis of parliamentary speeches linking climate change and mental health from 2001 to 2020 showed that most speeches made only general references to mental health and climate change, suggesting only a broad-scale acknowledgement among some decision makers of the potential connexion between these two domains. More specific references were particularly focused on flooding events and anxiety. Flooding is one of the main current and projected direct impacts of climate change on the UK, with one in six properties housing 5.2 million people at risk of flooding in England alone (31). Public Health England (PHE) reported significantly higher levels of anxiety in the aftermath of significant floods as part of the English National Study of Flooding in 2018 (9), and awareness of these issues appears to have reached a number of MPs, particularly those with affected constituents. The issue has also been raised by Lord Bethell (2019) in the House of Lords where the speakers are not speaking on behalf of a specific constituency. The 2018 PHE report also found flooding was linked to higher rates of post-traumatic stress disorder (PTSD) (9). Within parliament, however, there has been no acknowledgement of mental health impacts of substantial flooding other than anxiety over the past 20 years. With flooding events increasing in frequency in the UK, policy action is required to mitigate the risk and address the impacts on affected individuals and communities, including the full spectrum of mental health impacts.

Flooding is not the only climate-related risk that threatens the UK; other impacts will increase risk to current and future public mental health. In particular, extreme heat and heatwaves are predicted to increase. The record-equalling hot UK summer of 2018, for example, is expected to be an average UK summer by around 2050 (32). The impacts of these other climate events on mental health have been missed entirely in parliamentary debate. This is a notable omission given the strong evidence of the link between higher temperatures and suicides, hospitalisations for mental health disorders, worse population mental health and wellbeing, and increased physical impacts of heat, including higher mortality rates, for individuals with diagnosable mental health disorders (3, 33).

Newer planetary (mental) health terms used to describe the emotional toll of climate change, including eco-anxiety and ecological grief, were notably absent in parliamentary speeches, potentially signalling a lack of engagement with such experiences that are rapidly proliferating in public discourse, media and academic literature. Although these terms were not explicitly mentioned, parliamentarians did appear to reference indirect impacts of climate change on communities (31% of relevant speeches). While parliamentarians might not (yet) have adopted the terminology that is commonplace in academia and public media, evidence of some conceptual integration suggests a growing understanding that climate change evokes emotional responses, can engender distress and ultimately affects mental health. Nevertheless, in absolute terms, these links were uncommonly addressed in parliamentary speeches, indicating a relative disconnect from the experiences described by growing numbers of (particularly young) people and documented by academic researchers. It is important to note that government inaction and apparent failure on climate change is a key driver of high levels of distress and impacts on daily life and wellbeing experienced by young people (34). Distress has been found to be highest in countries with the least climate action (34), highlighting the need for any government serious about youth mental health to also show leadership in substantial and sufficient climate action.

Further, our analysis found speeches mainly focused on *adapting* to climate change rather than *mitigation* efforts. Debates among decision makers about how communities may adapt to protect themselves and their mental health from climate change are important, particularly as adaptation is often overlooked in policy and planning [e.g., see (35) for speeches of local-level consideration of mitigation and adaptation]. However, climate change mitigation arguably should be equally prioritised as a policy response to the documented mental health impacts, as this helps to minimise the impact of climate change (including on mental health) and reduce need for further and more extreme adaptive measures and mental health support required in the future. As suggested by Lawrance et al. (25), there are synergistic policy options that aim to simultaneously mitigate climate change and provide positive impacts for mental health. Examples include the reduction of air pollution, increasing accessibility of active transport options, or improving energy efficiency of housing. Through implementing these co-beneficial policies as a part of the UK government's plan to achieve Net Zero greenhouse gas emissions by 2050, the economic and social burdens of climate change, mental health and their combined effects could be reduced. Through tailoring and prioritising policies that promote positive mental health outcomes, maximum impact can be achieved with cost saving benefits for government and positive mental health benefits for the entire UK population.

Although we anticipate the findings of this study will be useful to guide efforts to drive appropriate responses to the challenges the UK faces in managing climate change and mental health policy and planning, there are some limitations. The low number of identified speeches necessitated a descriptive analysis on links between climate change and mental health. This has generated some preliminary insights into the key focal points for decision makers, while identifying conspicuous gaps. More in-depth approaches such as network analyses and more elaborate content analyses that engage with historical context may provide further insight into the contextual factors that underpin these speeches. Additionally, although our methodology did include the use of thematic analysis (which draws on qualitative methods) to create the categories of interest for quantifying frequency of mentions, and these categories are exemplified within the quotations, the analysis relies mainly on a quantitative approach. This was because the purpose of the paper was to provide the first top-level overview of whether this issue is being considered and the context in which it is being discussed. Therefore, this work could prompt further analysis of the nuance of the language used by parliamentarians on this issue using a more qualitative approach. Further, this research used a wide selection of keywords to search but it is conceivable that the analysis has missed some idiosyncratic and uncommon combinations of terms related to the issue. This is also a fast-moving and dynamic space, such that a repeat of this exercise will likely reveal new insights in how decision makers are adapting to advances in this area.

Another limitation of the findings in this paper is it focuses on how the links between climate change and mental health are being discussed within speeches by Members of Parliament (MPs) and the House of Lords. However, it is difficult to know how frequently these discussions are being made within the government but outside the formal speeches made in the Houses of Commons and Lords. It is also unclear how discussions ultimately influence legislation, and whether certain evidence, testimonials or narratives are more compelling and influential than others. Further research could use interviews of members of All-Party Parliamentary Groups and Select Committees to see if they have also had speeches on these topics and in what contexts. This could provide a fuller picture on awareness of the impact of climate change on mental health and the ways they are beginning to address this. Additionally, there is still limited evidence on how groups such as think tanks, medical professional bodies and advocates are discussing this issue. By asking these questions to all parties who have influence in this policy space, there is greater potential to ensure the correct and most productive conversations are taking place so that the potential impact of climate change on mental health can be mitigated.

Furthermore, as we have not looked at the impact of well designed, co-beneficial climate policies on mental health or indeed the impact of poorly designed policies in this study, there is also the opportunity to assess this impact *via* surveys or interviews with members of the public. Such research would provide a more holistic view of the issues and the true scale of the impact parliamentarians have on climate change-related mental health outcomes. Importantly, the evaluation of climate policy impacts on health and mental health will be an important part of any climate policy planning (36). Finally, climate change is an international crisis which cannot be tackled by one or a small minority of countries alone, and the impact of climate change on mental health is certainly not an issue isolated to the UK. Comparative studies with other countries where the impacts of climate change are more severe (e.g., Australia or the Philippines), or where multi-national responses are negotiated (e.g., EU) could aid in understanding the broader context for policy responses to the mental health impacts of climate change, and inform the UK response.

There appears to be a growing understanding of the links between climate change and mental health across sectors. For example, the Royal College of Psychiatrists recently released a position statement detailing the risks of climate change to mental health and how this should be tackled, indicating their desire to begin mitigating against the threat (37). The links between mental health and climate change have also recently been addressed in EU and UK briefing papers and discussed for the first time at COP. However, our analysis suggests there remains a gap in knowledge and awareness within parliament. Going forward, it is important that members of parliament and civil servants receive adequate briefing about the growing evidence base for the mental health emergency of the climate crisis, the significant costs of climate inaction for mental health, and opportunities for co-beneficial policies to simultaneously support a mentally healthier, safer climate future. Such awareness raising is a pre-requisite for adequate legislation to protect the mental health and wellbeing of the UK and global population in the face of a changing climate.

## Data availability statement

The raw data supporting the conclusions of this article will be made available by the authors, without undue reservation.

## Author contributions

All authors listed have made a substantial, direct, and intellectual contribution to the work and approved it for publication.

## Funding

EL's contribution to this project was partly enabled by the generous support of the Lenore England Innovation Fund at the Institute of Global Health Innovation.

## Conflict of interest

The authors declare that the research was conducted in the absence of any commercial or financial relationships that could be construed as a potential conflict of interest.

## Publisher's note

All claims expressed in this article are solely those of the authors and do not necessarily represent those of their affiliated organizations, or those of the publisher, the editors and the reviewers. Any product that may be evaluated in this article, or claim that may be made by its manufacturer, is not guaranteed or endorsed by the publisher.
